# Efficiency in COVID-19 Vaccination Campaigns—A Comparison across Germany’s Federal States

**DOI:** 10.3390/vaccines9070788

**Published:** 2021-07-14

**Authors:** Georg Götz, Daniel Herold, Phil-Adrian Klotz, Jan Thomas Schäfer

**Affiliations:** Department of Economics, Justus Liebig University Giessen, Licher Strasse 62, 35394 Giessen, Germany; georg.goetz@wirtschaft.uni-giessen.de (G.G.); phil.a.klotz@wirtschaft.uni-giessen.de (P.-A.K.); jan.t.schaefer@wirtschaft.uni-giessen.de (J.T.S.)

**Keywords:** COVID-19, vaccination campaign, efficiency

## Abstract

Vaccination programs are considered a central pillar of the efforts to stop COVID-19. However, vaccine doses are scarce and several organizational and logistical obstacles, such as the timing of and reserves for second shots and delivery failures, apparently slow down vaccination roll-outs in several countries. Moreover, it is an open question as to where vaccines are administered as efficiently as possible (vaccination centers, hospitals, doctor’s offices, pharmacists, etc.). The first aim of our study was to systematically evaluate the efficiency of a country’s vaccination campaign. The second aim was to analyze how the integration of doctors’ offices into a campaign that formerly relied only on vaccination centers affected the speed of that campaign. Using data on vaccine deliveries and vaccinations given in Germany, we find considerable differences across federal states in terms of efficiency, defined as the ability to administer the most vaccinations out of a given number of available doses. Back-of-the-envelope calculations for January to May 2021 show that vaccinations would have been 3.4–6.9% higher if all federal states had adopted a similar ratio between vaccinations given and vaccines stored, as the most efficient states did. This corresponds to 1.7–3.3% of Germany’s total population. In terms of our second research goal, we find evidence that the integration of doctors’ offices into the vaccination campaign significantly increased the ratio of vaccinations administered out of a given stock of vaccine doses. On average, there appears to be a structural break in this ratio after doctors’ offices were integrated into the vaccination campaign on 5 April 2021. On average, an additional 11.6 out of 100 available doses were administered each week compared to the period prior to that date. We conclude that there are considerable regional differences in the efficiency of the vaccination roll-out. Systematic efficiency analyses are one step to detecting inefficiencies and to identify best practices that can be adopted to eventually speed up the vaccination roll-out in a country.

## 1. Introduction

On 31 December 2019, the World Health Organization was informed of a new kind of infectious disease that was first identified in Wuhan, China [1]. It took only a few months for the virus, later known as SARS-CoV-2, to become a pandemic and spread across the globe [2,3]. By the time this paper was written, the total number of casualties was over 3.9 million worldwide, with over 180 million confirmed cases (data provided by WHO as of 28 June 2021, see https://covid19.who.int/, accessed on 28 June 2021).

Vaccination is considered a central pillar in the effort to stop the COVID-19 pandemic [4]. Despite the scarcity of vaccines, and subject to a prioritization of vulnerable and/or exposed individuals within the population [5,6], the policy goal is clear: roll out the available doses as quickly and efficiently as possible. However, the administrative and logistical challenges are substantial [7,8,9]. For instance, many countries report difficulties in the logistics of the vaccination roll-out, including unexpected delivery failures and the timing of second shots [10]. To overcome these obstacles, vaccine reserves are built. When reserves are too low, appointments have to be re-scheduled and no more shots can be given. When reserves are too high, more vaccinations could be given without compromising second shots. Vaccinations are at a sub-optimal level in both situations. It is important to detect and eventually avoid these inefficiencies that prolong the pandemic and cost lives [11,12,13]. This article analyzes this problem using Germany and its federal states as an example.

Another important aspect of the vaccination roll-out’s efficiency is the question of where the population can receive vaccinations. Potential candidates include vaccination centers, hospitals, retail pharmacies or doctors’ offices. The integration of general practitioners into a country’s vaccination campaign has been especially discussed in the literature, for instance, in terms of overcoming vaccine “hesitancy” in the population [14].

The vaccination roll-out in Germany started in December 2020. Even though every federal state faces essentially the same problem of determining the optimal level of vaccine reserves to maintain a smooth vaccination campaign, the 16 federal states show noticeable differences in the progress of their respective vaccination roll-outs. The vaccination campaign in Germany was based in vaccination centers until 5 April 2021, when doctors’ offices were officially integrated into the campaign (https://bit.ly/35PaZoi, accessed on 28 June 2021).

The aims of our study are two-fold. The first aim is to determine a measure of the efficiency of the federal states’ vaccination campaigns. Data Envelopment Analysis (DEA) is used to analyze relative efficiency by systematically comparing vaccine deliveries and stocks (input) with vaccinations given (output). Those federal states that are able to maintain a smooth vaccination campaign with the lowest vaccine reserves are identified as efficient. Efficiency is interpreted in relative terms, thus the most efficient federal states constitute a lower bound on efficiency. The benefits of decreasing inefficiencies can be approximated in a counterfactual scenario where it is assumed that every federal state adopts the ratio between vaccinations given to available doses as the most efficient states. The second aim of our study is to investigate the effect the integration of doctors’ offices had on Germany’s vaccination campaign. We do so using a fixed effects panel regression.

## 2. Materials and Methods

### 2.1. Dataset & Ethical Approval

Our dataset is comprised of two sources of data. Data on vaccine deliveries are available on the website of the Federal Ministry of Health, https://bit.ly/3vQAu3a (accessed on 28 June 2021). Data on daily vaccinations are published online by the Robert Koch Institute (RKI), https://impfdashboard.de/ (accessed on 28 June 2021). On the official website of the RKI, only recent data on vaccinations are available. However, historical data are made available via github.com by members of the German public broadcaster ARD, https://bit.ly/3vWPzQH (accessed on 28 June 2021). Our observation period was 27 December 2020 (when the first delivery arrived) to 16 May 2021.

The data used in this study are publicly available, highly aggregated and completely anonymized. We therefore consider this study exempt from ethical review.

In Germany, two mRNA-based vaccines produced by Biontech/Pfizer and Moderna and one vector-based vaccine produced by Astra-Zeneca were used. Towards the end of the observation period, a second vector-based vaccine produced by Johnson&Johnson was approved. According to contemporary guidelines in Germany, immunization with mRNA-based vaccines (and the vaccine produced by Astra-Zeneca) required two shots that had to be given within 6 weeks (12 weeks). Only one dose of the vaccine produced by Johnson & Johnson was required. Official guidelines were provided by the Federal Institute of Vaccines and Biomedicines (Paul Ehrlich Institute) (https://bit.ly/2Qud8le, accessed on 28 June 2021); an overview of the relevant information on storage requirements can be found, for example, here: https://bit.ly/2RmHy9J (accessed on 28 June 2021).

The data published by RKI are frequently revised ex post. Occasionally, RKI reported daily vaccinations of zero for some federal states. Missing vaccinations are apparently attributed to subsequent days by RKI. As a consequence, the data presented here might differ slightly from the aggregated figures published by RKI.

### 2.2. Method: DEA

A DEA is a method for comparing the relative efficiencies of different Decision Making Units (DMUs). The method was formalized by [15]. Since then, it has been used to analyze the performance of water [16] or electricity [17] suppliers as well as railroad firms [18]. In the health care sector, the method has been applied to hospitals [19,20] and also to vaccination centers [21]. The concept is closely related to cost-effectiveness and cost-utility analyses in health economics [22,23]. For an extensive review of the various applications and refinements of the technique, see [24].

In a DEA, efficiency is measured as a deterministic ratio between inputs and outputs. In the original formulation of [15], there are j=1,…,n DMUs with r=1,…,s outputs and k=1,…,m inputs. The known values of output *r* and input *k* of DMU *j* are denoted by $y_{rj}$ and $x_{kj}$, respectively. To find the most efficient among the *n* DMUs, the following program can be used:

(1) $$\begin{array}{ll} \underset{u_{r},v_{k}}{\max}\;\;\; & h_{0}=\frac{\sum_{r=1}^{s}u_{r}y_{r0}}{\sum_{k=1}^{m}v_{k}x_{k0}}\\ s.t. & \frac{\sum_{r=1}^{s}u_{r}y_{rj}}{\sum_{k=1}^{m}v_{k}x_{kj}}\leq 1\;\forall j=1,\ldots ,n,\\ & v_{r},u_{k}\geq 0\;\forall r=1,\ldots ,s,k=1,\ldots m. \end{array}$$

The weights $v_{r},u_{k}$ are endogenously determined by a comparison of all DMUs included as a reference. The problem can then be reformulated to yield a program that is solvable via linear programming. A detailed derivation can be found in [15] or in textbooks such as [25].

In addition to a DEA with constant returns to scale (CRS), DEAs were computed under the assumption of variable returns to scale (VRS). In a VRS DEA, the production possibility frontier is non-linear and defined by multiple DMUs. The concept of VRS DEA goes back to [26] and is sometimes also referred to as the BCC model. Note that the BCC model has a slightly different optimization problem; for a more detailed overview, see Chapter 2 in [25]. The question of whether CRS or VRS is assumed is especially important in applications in the health care sector [25], Ch. 16.4.4.2, especially when it comes to the analyses of vaccination centers [21].

The DEA is an appropriate method for analyzing the relative efficiency levels of the vaccination roll-out of Germany’s federal states for the following reasons. The DEA is a non-parametric method, that is, no assumptions on the functional form of the production function have to be imposed. Second, the DEA is used to analyze the relative performance of not-for-profit entities. In the case at hand, the overall policy goal is to maximize output, that is, to roll out as many vaccinations as possible given the available doses. This is in contrast to, for example, a profit maximizing firm that takes into account the price effects of its output choice.

Three types of models were computed with different output variables. In models T, 1S and 2S, the respective output variables are the total number of shots given, the total number of first shots given and the total number of second shots given in week *t*. The observation period for model 2S starts on 17 January 2021, the day the first second shot was recorded. Comparing the results of models 1S and 2S allows for an identification of the prioritization of federal states. A federal state that has high scores in model 2S but performs relatively poorly in model 1S can be considered to prioritize the full immunization of the population.

The input variable is the sum of vaccine deliveries in week *t* and vaccine reserves in week t−1. This variable approximates the amount of doses available for vaccination in week *t*. Even though this variable potentially overestimates the number of available doses in absolute terms (e.g., because doses arriving towards the end of week *t* might not be available for vaccination in that week), it does not systematically bias the comparison of the federal states because every state is affected in the same way (see the fluctuations in deliveries presented in Section 3.1 The results are robust to variations in the input variable. As an example, the results of DEAs computed with vaccine reserves in t−1 are presented in Appendix A.

In this study, separate DEAs were computed for each week. A federal state can then be considered efficient if it receives high scores in many periods. This was done because the scores of a single DEA might be biased, for example, due to large deliveries to a federal state (or the lack thereof) towards the end of the respective observation period, which can lead to relatively high (or low) inputs in relation to outputs compared to federal states that did not (or did) receive larger shipments. A further advantage of examining the results of multiple DEAs is that the results become more robust against outliers resulting from, for example, errors in the data or public holidays in some but not all federal states.

### 2.3. Method: Counterfactual Scenario

To illustrate the potential impact of improvements in efficiency on the progress of the vaccination campaign, a back-of-the-envelope calculation was carried out. A ratio of total vaccinations given in week *t* (output) to vaccine deliveries in week *t* and vaccine reserves at the end of week t−1 (input) was determined. Formally, federal state *i*’s share in week *t* reads:

(2) $$s_{i,t}=\frac{{\mathrm{vaccinations}}_{i,t}}{{\mathrm{deliveries}}_{i,t}+{\mathrm{reserves}}_{i,t-1}}.$$

For example, $s_{i,t}=0.6$ would indicate that in federal state *i* in week *t*, 60% of the doses available for vaccination in week *t* are actually administered, whereas the remaining 40% are held back as reserves.

The following counterfactual scenario was assumed to compute the potential gain in vaccinations administered when efficiency is improved. Suppose federal states *k* and *l* are identified as the most and second most efficient federal states, respectively, by the DEA described in Section 2.2. In the counterfactual analysis, it was assumed that each federal state that is identified as inefficient by the DEA adopts the ratio of vaccinations given to available doses in *k* in each week *t*. Formally, $s_{i,t}=s_{k,t}$ for all *i* and *t*. A more conservative perspective was taken by assuming that each federal state, except for *k*, adopts the ratio of the second most efficient federal state *l*, $s_{i,t}=s_{l,t}$ for all i≠k and *t*.

### 2.4. Method: Vaccination at Doctor’s Offices

Based on the data, it was tested whether there was a structural break in $s_{i,t}$, as defined in Equation (2), after 5 April 2021, when general practitioners were integrated into Germany’s vaccination campaign. In doing so, the following fixed effects panel regression was estimated:

(3) $$s_{i,t}=\alpha +\beta t+\delta D_{\mathrm{April}\;5}+D_{i}+\epsilon_{i,t}.$$

The dependant variable in Equation (3) is the share $s_{i,t}$. Note that this is the ratio between (unweighted) output and input of DEA model T (see above). The observed shares are depicted in Section 3.1 for each federal state.

In Equation (3), $s_{i,t}$ is explained by a time trend *t*, the variable $D_{\mathrm{April}\;5}$ takes the value 1 for the period after 5 April 2021 and 0 otherwise, as well as a federal state specific fixed effect $D_{i}$. The latter controls for time-invariant effects specific to a federal state. This approach allows for an investigation of whether the integration of doctors’ offices into the German vaccination campaign potentially improved the efficiency of the campaign on average.

## 3. Results

### 3.1. Results: Descriptive Statistics

**Figure 1:**
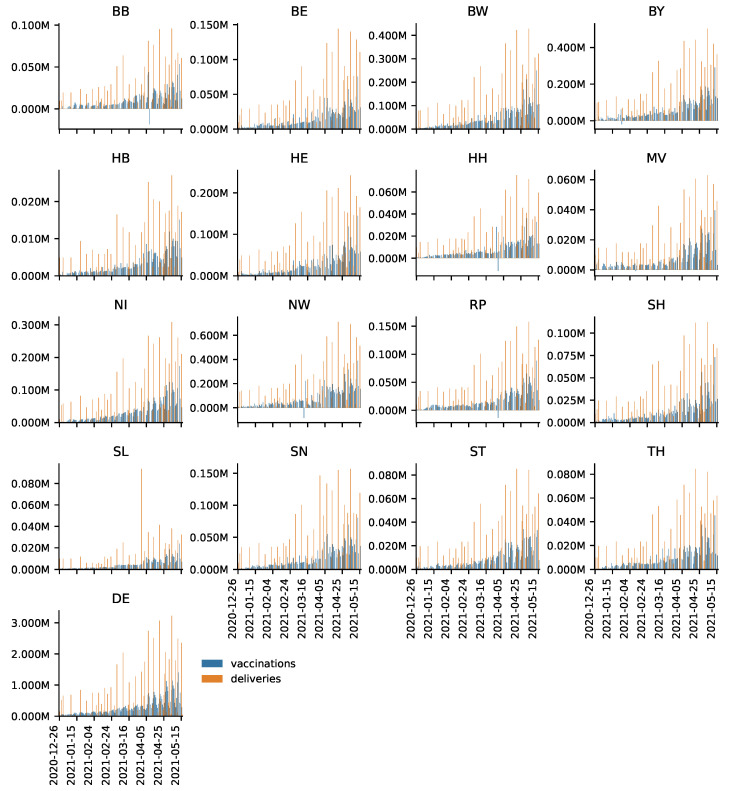
Daily number of people vaccinated and deliveries by Federal State.

In Figure 1, daily vaccine deliveries and vaccines administered are depicted for each federal state as well as for Germany as a whole (DE) for the period from 27 December 2020 to 16 May 2021. One can see that deliveries are relatively infrequent and their magnitude varies. This corroborates the organizational challenge of the vaccination campaign, especially against the background that it is necessary to administer two shots to achieve full immunization. Remarkably, Saarland (SL) received a delivery of over 80,000 vaccines at the end of March. These deliveries, however, seem to have barely affected contemporary vaccinations. Given that Saarland continued to receive relatively high deliveries in subsequent weeks, this indicates that reserves built up. Recall that observations of zero or even negative vaccinations administered stem from errors in the data, which are corrected by RKI ex post.

Table 1 presents an overview of total deliveries, vaccinations broken down by first and second shots, and storage quotas for the first quarter of 2021. The storage quota relates deliveries to total vaccinations. For instance, Bremen (HB) had 6164 doses in stock, which was approximately 4.66% of total deliveries. Note that Table 1 provides a snapshot: storage quotas are inflated in some federal states (e.g., Saarland (SL); see above) that received large shipments by the end of the first quarter whereas others did not.

**Figure 2:**
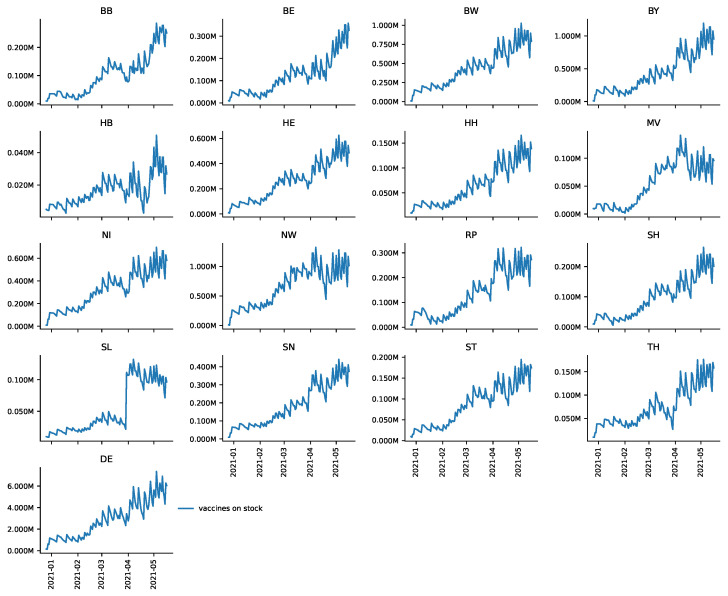
Daily number of vaccines in stock by Federal State.

Figure 2 presents vaccine reserves broken down by federal states for the period from 27 December to 16 May 2021. For Germany as a whole (DE), towards the end of the observation period there were almost 6 million doses in stock. This constitutes jabs for over 7% of Germany’s population (83,190,556 people as of 30 September 2020, based on information provided by the Federal Statistical Office, https://bit.ly/3bOABoR, accessed on 28 June 2021). One can see a sawtooth-like shape of deliveries and vaccinations given; however, the level of reserves drastically increases over time. Even though some federal states, such as Bremen (HB), apparently reduced vaccine reserves to some degree during the observation period towards mid May, every federal state seems to have built up substantial vaccine reserves. Again, daily data have to be interpreted with care due to the ex-post revisions of RKI.

Finally, Table 2 presents descriptive statistics on reserves per first doses given. For instance, in Brandenburg, an average of 0.47 doses were held as reserves per first shot given with a median of 0.39. Consistent with the observations described above, Bremen shows relatively low median reserves whereas federal states, such as Saarland (SL) and Lower-Saxony (NI), apparently had relatively high median reserves.

**Figure 3:**
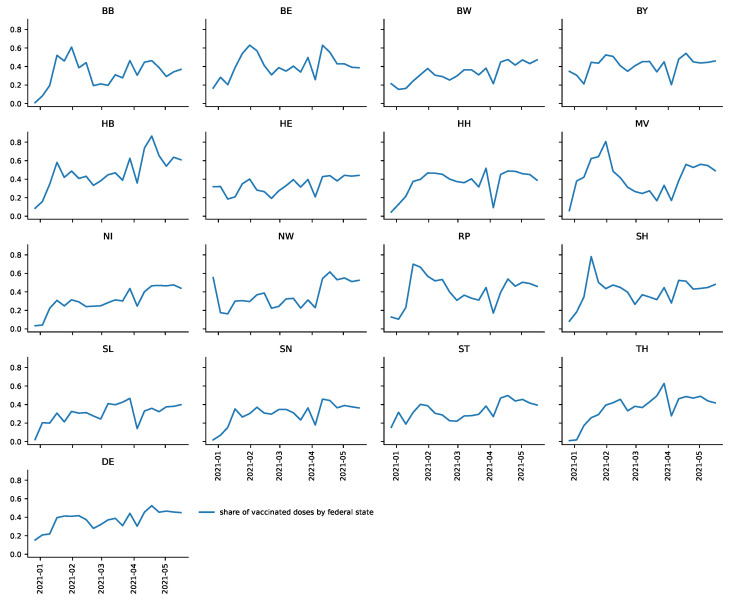
The share of vaccinations given in week *t* in relation to deliveries in week *t* and reserves in week t−1$\left(s_{i,t}=\frac{{\mathrm{vaccination}}_{i,t}}{{\mathrm{deliveries}}_{i,t}+{\mathrm{reserves}}_{i,t-1}}\right)$ for all federal states.

Figure 3 illustrates that $s_{i,t}$ has substantial fluctuations over time in most federal states and differs remarkably between them. For instance, in Bremen (HB), that share relatively quickly increases to values over 0.4 and even jumps to over 0.8, whereas in Saarland (SL) the share never exceeds 0.5.

### 3.2. Results: DEA

Table 3 presents the results of the mean DEA scores in Models T, 1S and 2S of DEAs performed for each week of our observation period. Here, constant returns to scale (CRS) were assumed.

Table 3 shows that Bremen was assigned the highest average DEA score with 0.8289 in model T, 0.7842 in model 1S and 0.689 in model 2S. This indicates that Bremen (HB) had the most efficient vaccination roll-out in Germany under the CRS assumption.

One might argue that the results of a small federal state, such as Bremen with a total population of less than 600,000, are not applicable to larger federal states such as Bavaria (BY) or Northrhine-Westphalia (NW) with populations of 13.8 million and almost 18 million, respectively, and a lower population density. Likewise, Bremen receives fewer vaccine deliveries (see above), which potentially eases the organizational burden of the vaccination roll-out. In other words, a vaccination campaign might be susceptible to decreasing returns to scale so that it becomes increasingly difficult to distribute vaccinations the larger the input of vaccines. Thus, the scores of VRS DEAs for each week of the observation period were computed. The average scores are presented in Table 4.

The results presented in Table 4 show that Bremen is assigned average DEA scores of 1 in every model. This means that Bremen is among the federal states that define the production possibility for every week. In contrast to the CRS DEA, larger federal states, such as North Rhine-Westphalia, were assigned significantly higher DEA scores.

Potential drivers of the different levels of efficiency are explained in Section 4. Appendix A contains a more thorough discussion of the results of the DEA as well as a robustness check.

The results presented in Table 3 and Table 4 indicate differences in the prioritization of first and second shots between federal states. Apparently, federal states with lower scores in model 1S than in model 2S (e.g., Saxony, SN) focus on full immunization of the population whereas federal states with lower scores in model 2S than in model 1S (e.g., Lower Saxony, NI) seem to prioritize first shots.

### 3.3. Result: Counterfactual Scenario

Assuming that all federal states adopt the ratio (2) for Bremen ($s_{\mathrm{HB},t}$) or North Rhine-Westphalia ($s_{\mathrm{NW},t}$) in each week *t*, we compute hypothetical vaccinations given per federal state. The results are presented in Table 5.

The results presented in Table 5 indicate that over 2.7 million (+6.85%) more vaccinations would have been given until 16 May 2021 if all federal states had adopted $s_{\mathrm{HB},t}$ in each week *t*. This corresponds to 3.29% of the German population.

A comparison with North Rhine-Westphalia, whose vaccination roll-out is remarkably efficient given the size of the federal state, yielded less optimistic, yet noticeable results. According to the figures presented in Table 5, almost 1.4 million more doses would have been administered if all federal states (except for Bremen) had adopted North Rhine-Westphalia’s ratio between vaccinations given and reserves and deliveries. This still corresponds to 1.65% of the entire population and constitutes a plus of around 3.44%.

### 3.4. Results: Vaccination at Doctor’s Offices

The results of the fixed effects panel regression Equation (3) can be found in Table 6.

The results presented in Table 6 indicate that there was a statistically significant structural break on 5 April 2021, for Germany. That is, $s_{i,t}$ increased by 11.6% on average in the period after 5 April 2021, compared to the period prior to that date. In other words, the share of doses held back as reserves decreased by the same fraction on average. This means that, on average, 11.6 more out of 100 available doses—measured by vaccine reserves plus vaccine deliveries—were given each week.

The results indicate that the structural break diagnosed on average for Germany seems to be driven by some federal states that exhibit a relatively strong structural break (e.g., NW). Not every federal state shows a statistically significant structural break in the period after 5 April 2021. Moreover, on average, based on the results presented in Table 6, no statistically significant time trend was diagnosed. These findings are supported by the various robustness checks presented in Appendix B. These findings not only capture the effect of the integration of doctors’ offices into the vaccination campaign as is discussed in Section 4.

## 4. Discussion

The primary aim of the present study was to determine a lower bound on efficiency in the German federal states’ vaccination campaigns. Several DEAs were performed and Bremen apparently defines the efficiency frontier during the observation period. Among the larger federal states, North Rhine-Westphalia receives remarkably high efficiency scores. With VRS, this federal state has relatively high average efficiency scores, whereas under CRS its average scores are relatively low.

A counterfactual scenario was computed based on a back-of-the-envelope calculation. It was shown that an increase in efficiency could have led to an increase in vaccinations in the magnitude of 3.44–6.85%, which corresponds to 1.65–3.29% of the German population. This shows that analyses of the efficiency of vaccination roll-outs can play an integral role in overcoming the COVID-19 pandemic. Avoiding excessive reserves is crucial—a vaccine that is unused cannot save lives. That countries handle the vaccine doses available to them as efficiently as possible seems to be particularly important against the background of pronounced vaccine scarcities in low-income countries (e.g., [27]).

By the time this paper was written, 53% (34.5%) of the German population had received a first (second) shot. In comparison to the other 30 countries of the EU/EEA, Germany ranks eighth (sixth) when it comes to first (second) shots. The German vaccination campaign was slower compared to some non-EU countries: the United States (first shots: 53.5%, second shots: 45.5%), Canada (67.4%, 25.6%), Israel (64%, 59.6%) and the United Kingdom (65%, 47%) (see https://ourworldindata.org/covid-vaccinations, accessed on 28 June 2021). In the literature, organizational and country-specific factors that influence the speed and efficiency of the COVID-19 vaccination roll-out are identified. Potential drivers of the high speed of Israel’s vaccination campaign include the small size of the country both in terms of area and population, a relatively young population, an efficient health care system with IT-heavy organization, large vaccine orders and a clear prioritization system for vaccinations within the population in the early phases of the distribution process [28,29]. In particular, Israel relaxed its prioritization system at some point to avoid diminishing returns [30]. A similar strategy was pursued in the USA [31]. There is also evidence that the use of online communication effectively increased the speed of the United States’ vaccination campaign [31,32]. In contrast to that, for example in the UK, appointments were initially allocated mostly by text messages or mobile phone calls. The vaccination campaign in the UK was sped up when officials decided—as one of the first countries worldwide—to extend the interval between first and second shots of two important vaccines in order to vaccinate as many people as possible at least once [33].

Given the little information that is publicly available regarding the administrative processes of the vaccination roll-out in the different federal states, it is difficult to pinpoint certain aspects where the federal states’ organization of the vaccination roll-out differs. Possible explanations for the observed differences in efficiency include diverging practices when it comes to building reserves to, for example, avoid the re-scheduling of future appointments (https://bit.ly/2RoBAoO; all links in this section accessed on 28 June 2021), the handling of appointments for the different priority groups (https://bit.ly/3ob1Leu), the use of more efficient syringes (https://bit.ly/3hyfDyj) and the administration of appointments (https://bit.ly/3tFoi4h).

According to the results presented in Section 3.2, Bremen’s vaccination campaign is apparently the most efficient. It is remarkable that the state’s vaccination campaign was not solely planned and executed by Bremen’s government. Local firms have supported the campaign by establishing a vaccination initiative (“Bremen impft”, https://bit.ly/3x1JmVd). This collaboration between private firms and public officials is responsible for the administration of the—by the contemporary standards as of March 2021—largest vaccination center in Germany (https://bit.ly/3gWP05b). Moreover, it is documented that there was close cooperation with health insurance companies to systematically identify and allocate appointments to high-risk patients in Bremen (https://bit.ly/3y1Bi6Y).

The second aim of this study was to analyze the effect that the integration of general practitioners into Germany’s vaccination campaign on 5 April 2021 had on efficiency. The results indicate that there was a structural break for Germany as a whole in the period after that integration. On average, 11.6 more out of 100 available doses were vaccinated per week compared to the period before 5 April. This indicates that the integration of doctors’ offices into the campaign has sped up the vaccination roll-out.

While in other countries, such as the UK, general practitioners have been part of the vaccination campaign from the beginning (https://nyti.ms/2UOTi6h), in Germany, doctors’ offices were officially integrated into the campaign roughly four months after the beginning of the vaccination roll-out. Approximately 35,000 general practitioners started to administer COVID-19 vaccinations in the week after Easter in Germany. With around 102,000 doctors’ offices in Germany, this is around one third (https://bit.ly/35Y3748). In total, practitioners have ordered 1.4 million doses for vaccination in calendar week 14 (https://bit.ly/3x2yLcw). This number has continuously increased in subsequent weeks. Based on the dataset used in this paper, deliveries to doctors’ offices were around 2.5 million doses in calendar week 19, on a par with those to vaccination centers.

General practitioners order the desired number of vaccine doses for week t+1 until Tuesday of week *t* at local pharmacies. The orders of each federal state are centrally monitored to ensure a fair allocation of doses across Germany. Physicians are informed on Thursday of week *t* about how many doses they will receive in week t+1. Appointments are scheduled at the general practitioner’s discretion (see https://bit.ly/3qEHPSB). Practitioners were officially constrained by the prioritization system until 7 June 2021, when the system was abandoned (see https://bit.ly/3qxJPMs). Administering COVID-19 vaccinations is associated with a relatively large bureaucratic burden on general physicians, which is considered especially cumbersome because those vaccinations are an extra service offered by practitioners [34].

The results of the present paper have implications for clinical management. First, expectation management seems to be important. An efficient vaccination roll-out with as little reserves as possible can make it necessary to communicate that appointments (especially for first shots) might be re-scheduled in case of delivery disruptions. Second, integrating doctors’ offices into a vaccination campaign can apparently speed up a vaccination roll-out that is otherwise based on vaccination centers. Third, the results above indicate that the more efficient management of appointments (reserves for second shots, (re-)scheduling, identification of vulnerable individuals) can be considered a way to improve efficiency. The German vaccination campaign appears to be characterized by a high degree of bureaucracy. Even though further research is necessary, more flexible, innovative and IT-based solutions can be expected to speed up the vaccination roll-out.

The present study has some limitations. First, it constitutes a rather high level approach. A lower bound on efficiency, rather than an optimal inventory management, was determined. The latter could be analyzed, for example, in an (s,S)-model [35]. Such an approach requires a more sophisticated dataset including, for instance, information about planned vaccine deliveries. Moreover, the decision makers’ expectations have to be accounted for and the trade-off between first and second shots has to be discussed [36]. Second, the study at hand suffers from a lack of information about the administrative details and the causes of the differences in detected efficiency levels. The DEAs could also be enriched by more detailed information about health care workers, the number of vaccination centers, demographic and geographic factors, and so forth. Third, based on the data available to the authors by the time this paper was written it was impossible to analyze whether the vaccination of the most vulnerable individuals in terms of their risk of mortality was actually prioritized. Fourth, systematic, historical data on vaccine deliveries to doctors’ offices and to vaccination centers per federal state were also not available to the authors. This study therefore relied on a statistical analysis of the time series of vaccinations given and the available doses. As such, the structural break we identified in Section 3.2 might not only be driven by doctor’s offices being integrated into the vaccination campaign. It cannot be excluded that other factors also affected $s_{i,t}$ (see Equation (2)) and influenced the results. For instance, one of these factors could be the findings of [37] that a 12-week, rather than a shorter, timespan between first and second shots of the vaccine produced by Astra-Zeneca does not reduce protection against COVID-19. This might have led decision makers to reduce stock holdings of Astra-Zeneca’s vaccine, that is, all else being equal, leading to an increase in $s_{i,t}$. If this happened (with some delay) in the period after 5 April 2021, the effect of the integration of doctors’ offices into the vaccination campaign would be overestimated.

Most of the above limitations stem from a lack of appropriate data. If these data become available in the future, further analyses on the topic will be fruitful.

## 5. Conclusions

This study is a first attempt to systematically analyze the efficiency of the COVID-19 vaccination roll-out in different regions of a country. This exercise allows one to identify a lower bound of efficiency of that country’s vaccination campaign. Similar analyses can be performed for other countries as well, especially because data requirements are minimal. We used Germany as an example. Our findings indicate that efficiency comparisons, such as DEA, can be valuable for detect ing inefficiencies in a vaccination roll-out. Our results on the effect of integrating general practitioners into the vaccination campaign indicate an important avenue for how the administration of vaccinations might be sped up.

## Figures and Tables

**Table 1:** Summary statistics for q1.2021 by Federal State.

Federal State	Deliveries	Total Vaccinated	First	Full	Storage Quota
HB	132,255	126,091	90,234	35,857	4.66%
TH	432,150	403,299	280,682	122,617	6.68%
BE	697,200	635,103	427,101	208,002	8.91%
BB	472,590	429,998	328,564	101,434	9.01%
SH	550,575	494,930	378,776	116,154	10.11%
NI	1,503,825	1,328,867	920,694	408,173	11.63%
DE	15,974,175	14,005,686	9,763,805	4,241,881	12.32%
HH	347,325	301,837	211,080	90,757	13.10%
BY	2,569,635	2,232,881	1,516,433	716,448	13.11%
BW	2,085,075	1,798,007	1,253,952	544,055	13.77%
HE	1,188,420	1,022,298	691,217	331,081	13.98%
RP	790,185	676,868	495,688	181,180	14.34%
ST	418,200	351,265	251,865	99,400	16.01%
NW	3,363,300	2,739,983	1,913,865	826,118	18.53%
MV	300,225	234,839	159,250	75,589	21.78%
SN	856,875	654,525	405,999	248,526	23.61%
SL	266,340	176,290	129,287	47,003	33.81%

**Table 2:** Summary statistics on reserves per first dose given for the period from 11 January 2021 to 16 May by Federal State. The first two weeks of the campaign were left out here due to the low number of vaccinations in relation to deliveries.

Index	#Weeks	Mean	Std	Min	25%	50%	75%	Max
BB	18	0.47	0.22	0.24	0.31	0.39	0.58	0.88
BE	18	0.35	0.14	0.16	0.25	0.32	0.43	0.68
BW	18	0.56	0.32	0.2	0.28	0.5	0.76	1.34
BY	18	0.35	0.12	0.19	0.24	0.33	0.41	0.55
DE	18	0.42	0.17	0.2	0.24	0.42	0.51	0.7
HB	18	0.28	0.16	0.03	0.13	0.29	0.41	0.55
HE	18	0.56	0.28	0.21	0.3	0.51	0.78	1.05
HH	18	0.39	0.16	0.2	0.22	0.4	0.43	0.82
MV	18	0.36	0.21	0.07	0.18	0.26	0.56	0.68
NI	18	0.62	0.34	0.19	0.28	0.59	0.92	1.12
NW	18	0.54	0.3	0.14	0.19	0.59	0.75	1.08
RP	18	0.31	0.11	0.17	0.21	0.27	0.41	0.51
SH	18	0.31	0.12	0.15	0.23	0.26	0.4	0.56
SL	18	0.58	0.29	0.21	0.34	0.53	0.81	1.2
SN	18	0.58	0.25	0.28	0.35	0.55	0.74	1.12
ST	18	0.52	0.24	0.21	0.26	0.55	0.69	0.9
TH	18	0.46	0.35	0.13	0.23	0.38	0.51	1.55

**Table 3:** Average efficiency scores for the period from 27 December 2020 to 16 May 2021 for DEAs performed on a weekly basis under the CRS assumption.

Federal State	T	1S	2S
BB	0.5699	0.5248	0.4321
BE	0.7195	0.6708	0.6857
BW	0.5891	0.584	0.4581
BY	0.7371	0.7276	0.5477
HB	0.8289	0.7842	0.689
HE	0.5993	0.597	0.4873
HH	0.6495	0.643	0.4942
MV	0.7165	0.6591	0.4911
NI	0.5442	0.5483	0.3971
NW	0.6428	0.6554	0.4438
RP	0.7113	0.6455	0.5263
SH	0.7125	0.6531	0.5363
SL	0.5527	0.5643	0.4078
SN	0.5321	0.4814	0.5457
ST	0.5898	0.5712	0.4695
TH	0.6544	0.6155	0.6566

**Table 4:** Weekly average DEA efficiency scores for the period from 27 December 2020 to 16 May 2021 under the VRS assumption.

Federal State	T	1S	2S
BB	0.6487	0.6077	0.5159
BE	0.8042	0.7375	0.7332
BW	0.7346	0.7397	0.6222
BY	0.9408	0.9067	0.7495
HB	1	1	1
HE	0.6994	0.6813	0.5605
HH	0.7185	0.7187	0.5909
MV	0.7887	0.7602	0.6215
NI	0.682	0.6771	0.4849
NW	0.9793	0.9347	0.8258
RP	0.8004	0.7317	0.6078
SH	0.7873	0.7404	0.6273
SL	0.6446	0.6662	0.5935
SN	0.6071	0.5433	0.5984
ST	0.6499	0.6329	0.5401
TH	0.7277	0.6807	0.7274

**Table 5:** Counterfactual scenario where it is assumed that less efficient federal states adopt $s_{i,t}$ for i=Bremen or i = North Rhine-Westphalia every week *t*.

		Bremen		Northrhine-Westphalia	
Federal State	Act. Vacc	Hyp. Vacc	%-Gain	Hyp. Vacc	%-Gain
BB	1,114,386	1,273,349	14.26%	1,232,573	10.61%
BE	1,703,655	1,881,650	10.45%	1,819,474	6.80%
BW	5,268,410	5,630,827	6.88%	5,449,360	3.43%
BY	6,368,489	6,813,909	6.99%	6,594,566	3.55%
HB	357,057	357,057	0	357,057	0%
HE	2,967,320	3,213,995	8.31%	3,110,320	4.82%
HH	854,467	927,992	8.60%	898,988	5.21%
MV	794,509	825,545	3.91%	798,212	0.47%
NI	3,817,325	4,089,342	7.13%	3,955,702	3.62%
NW	8,869,664	9,171,453	3.40%	8,869,664	0%
RP	1,970,369	2,086,120	5.87%	2,018,964	2.47%
SH	1,396,932	1,486,886	6.44%	1,438,825	3.00%
SL	523,240	582,246	11.28%	565,857	8.14%
SN	1,912,294	2,144,315	12.13%	2,079,466	8.74%
ST	1,024,732	1,120,043	9.30%	1,084,781	5.86%
TH	1,041,879	1,120,897	7.58%	1,086,081	4.24%
Total	39,984,728	42,725,627	6.85%	41,359,888	3.44%

**Table 6:** Output table for Equation (3).

	Dep. Var. $s_{i,t}$	
$D_{\mathrm{April}\;5}$	0.116 **	(4.08)
Time trend	0.000941	(0.35)
Constant	0.333 ***	(13.43)
Observations	320	
$R^{2}$	0.348	
$R^{2}$ adjusted	0.311	

*t* statistics in parentheses; two and three asterisks correspond to p<0.001 and p<0.01, respectively.

## Data Availability

The data used in our study are publicly available on the website of the Federal Ministry of Health (https://www.bundesgesundheitsministerium.de/coronavirus/faq-covid-19-impfung.html; accessed on 28 June 2021), the Robert-Koch-Insitute (RKI) (https://impfdashboard.de/; accessed on 28 June 2021) and Github (https://github.com/ard-data/2020-rki-impf-archive/tree/master/data/9_csv_v2; accessed on 28 June 2021).

## Abbreviations

The following abbreviations are used in this manuscript:

RKI	Robert-Koch-Institute
DEA	Data Envelopment Analysis
DMU	Decision making unit
CRS	Constant returns to scale
VRS	Variable returns to scale
BCC	Banker, Charnes and Cooper
DE	Germany
HB	Bremen
TH	Thuringia
BE	Berlin
BB	Brandenburg
SH	Schleswig-Holstein
NI	Lower Saxony
HH	Hamburg
BY	Bavaria
BW	Baden-Wuertemberg
HE	Hesse
RP	Rhineland Palatinate
ST	Saxony-Anhalt
NW	Northrhine-Westphalia
MV	Mecklenburg Western Pomerania
SN	Saxony
SL	Saarland

## Appendix A. DEA

The influence of diminishing returns to scale was controlled for in the VRS DEA. However, there are further factors potentially affecting the results.

It is documented that there were occasional failures to deliver vaccines to certain areas (https://bit.ly/3uGEIul; accessed on 28 June 2021). Even though these are out of the control of the decision makers, it is unlikely that these delivery failures to individual federal states systematically affects the DEA scores over a period of over 4 months. The same holds for large-scale delivery failures of the vaccine of Astra-Zeneca (https://bit.ly/3jok2oe; accessed on 28 June 2021)). Moreover, the delivery failure of Astra-Zeneca’s vaccine affected Germany as a whole so that it should not affect the relative efficiencies of the federal states’ vaccination roll-outs.

One could argue that it is more difficult for the elderly to make and keep their appointments. This means that the broader the range of age cohorts eligible of being vaccinated the more strongly this could affect the DEA scores because, e.g., it requires more effort to vaccinate the elderly. In the first quarter the majority of vaccinations were given to the groups with highest (aged 80+) and second highest (aged 70–79) priority. Note that in the official regulation (“CoronaImpfV”) priority groups within the population are not only defined by age but also by other factors, such as prior diseases, social relevance or exposition to infected people. The official regulation can be found here: https://bit.ly/2TbIpdR (accessed on 28 June 2021). It does not appear to be the case that demographics largely impact our results. Despite some overlaps, the federal states’ age profiles (see https://bit.ly/349YNhb; accessed on 28 June 2021) do not seem to affect the results of our DEA. The following example illustrates this. If demographics had a significant impact on the relative success of the federal states’ vaccination roll-outs, federal states with a younger population such as Hamburg (HH) with an average age of 42.1 would have an advantage over those with an older population such as Thuringia (TH) with an average age of 47.4 years. Hamburg and Thuringia have about the same population while Hamburg is significantly smaller in terms of area. However, according to the results presented in Table 3 and Table 4 Hamburg is not unambiguously more efficient than Thuringia.

By the same token, our results do not seem to be largely driven by the federal states’ geography. For instance, Bremen (HB), the most efficient federal state, and Saarland (SL), among the federal states with the lowest efficiency scores, are both relatively small federal states. However, the scores of the federal states with the lowest population density, Mecklenburg Western Pomerania (MV) and Brandenburg (BB) might be driven by the countries’ geographies at least to some extent. Note that Mecklenburg Western Pomerania is assigned relatively high scores in model T (see Table 1 and Table 2 in the main text), which means that the federal state performs relatively well despite its dispersed population, anyway. In these countries one would expect that it is relatively difficult for people in rural areas to reach vaccination centers and that doctor’s offices are more efficient distributors of vaccination than large vaccination centers. Indeed, our analyses presented in Section 3.4 provide evidence that the integration of doctor’s offices into those country’s vaccination campaigns might have substantially increased efficiency. A fruitful extension would be to cluster the federal states. This would allow the researcher to identify the most suitable candidates for benchmarking.

A DEA with a different input is presented to demonstrate the robustness of the results presented in the main text. Table A1 and Table A2 present the average DEA scores of CRS and VRS DEAs computed for the period 27 December 2020 to 16 May 2021 with only vaccine reserves in week t−1 as the input variable (rather than vaccine reserves in t−1 plus vaccine deliveries in week *t*). The results remain largely unchanged.

**Table A1:** Average efficiency scores for the period 27 December 2020 to 16 May 2021 for DEAs performed on a weekly basis under the CRS assumption using vaccine reserves at the end of the previous week as the input variable. Models T, 1S and 2S use total, first and second shots given as the respective output variable.

Federal State	T	1S	2S
BB	0.4286	0.3988	0.3399
BE	0.5822	0.562	0.6169
BW	0.4186	0.4276	0.3587
BY	0.609	0.6221	0.5104
HB	0.7961	0.7846	0.7055
HE	0.4081	0.4175	0.3945
HH	0.509	0.5238	0.4301
MV	0.6064	0.5613	0.4875
NI	0.3868	0.4055	0.3063
NW	0.4773	0.5112	0.3465
RP	0.6069	0.5752	0.4555
SH	0.5925	0.5711	0.43
SL	0.3999	0.4243	0.3289
SN	0.3788	0.3543	0.4116
ST	0.3934	0.3979	0.353
TH	0.5383	0.5257	0.5536

**Table A2:** Weekly average DEA efficiency scores for the period 27 December 2020 to 16 May 2021 under the VRS assumption using the vaccine stock at the end of the previous week as the input variable. Models T, 1S and 2S uses total, first and second shots given as the respective output variable.

Federal State	T	1S	2S
BB	0.5351	0.513	0.4278
BE	0.7466	0.7067	0.7372
BW	0.6372	0.6644	0.5538
BY	0.9306	0.902	0.7484
HB	0.9765	0.9765	0.9753
HE	0.5624	0.558	0.4916
HH	0.6115	0.6341	0.5095
MV	0.7152	0.7076	0.5812
NI	0.588	0.5977	0.4124
NW	1	0.947	0.8212
RP	0.7748	0.7257	0.6129
SH	0.7314	0.7187	0.5911
SL	0.4928	0.5245	0.4633
SN	0.4922	0.4505	0.5259
ST	0.4948	0.5052	0.4366
TH	0.643	0.6179	0.6849

## Appendix B. Estimations

In the remainder of the Appendix, additional information to Section 3.4 are presented. First, we present an alternative estimation to analyze the effect of the integration of doctor’s offices into Germany’s vaccination campaign. The following fixed effects regression is estimated:

(A1) $$s_{i,t}=\alpha +\beta t+\gamma_{i}D_{i}\times t+\delta D_{\mathrm{April}\;5}+D_{i}+\epsilon_{i,t}.$$

In Equation (A1), the share $s_{i,t}$ is explained by a time trend, a federal state dummy $D_{i}$ interacted with the time trend and the dummy variable $D_{\mathrm{April}\;5}$ which takes the value 1 in the period starting on 5 April 2021 and 0 otherwise. Federal state-specific fixed effects are denoted $D_{i}$. With this specification, it is possible to disentangle how the share of vaccinations given in week *t* in relation to deliveries in week *t* and reserves in week t−1 evolves over time in the different federal states. The fixed effects are chosen such that the effect of the interaction term $D_{i}\times t$ has to be interpreted relative to the federal state Bremen (HB), as will be explained in more detail below. The results are presented in Table A3.

The results indicate that the share $s_{i,t}$ is significantly higher (1%-level) in the period after 5 April, which means that, on average, there is a structural break. The order of magnitude of the effect is the same as that presented in the main text. Note that this effect can be driven by doctor’s offices being integrated into Germany’s vaccination campaign, even though we cannot exclude that other factors also affected the results (see the discussion in the main text).

According to the results presented in Table A3 the share $s_{i,t}$ evolves over time on average. Every week, the share $s_{i,t}$ increases by 0.0108 plus the coefficient of the federal state dummy interacted with the time trend. This follows from $\frac{\partial s_{i,t}}{\partial t}=\beta +\gamma_{i}D_{i}$ based on Equation (A1). For instance, e.g., in Brandenburg (BB) the share of vaccinations given in week *t* in relation to deliveries in week *t* and reserves in week t−1 decreases by 0.0157−0.0108=0.0049 per week (see Figure 3 in the main text).

**Table A3:** Output table for Equation (A1).

	Dep. Var. $s_{i,t}$	
$D_{\mathrm{April}\;5}$	0.116 **	(3.98)
Time trend	0.0108 ***	(5.86)
BB × Time trend	−0.0157 ***	($-3.17\times 10^{14}$)
BE × Time trend	−0.0152 ***	($-5.25\times 10^{14}$)
BW × Time trend	−0.00465 ***	($-1.62\times 10^{14}$)
BY × Time trend	−0.0138 ***	($-4.37\times 10^{14}$)
HE × Time trend	−0.00836 ***	($-1.58\times 10^{14}$)
HH × Time trend	−0.0111 ***	($-3.27\times 10^{14}$)
MV × Time trend	−0.0209 ***	($-4.46\times 10^{14}$)
NI × Time trend	−0.00235 ***	($-8.04\times 10^{13}$)
NW × Time trend	−0.000310 ***	($-1.08\times 10^{13}$)
RP × Time trend	−0.0168 ***	($-4.15\times 10^{14}$)
SH × Time trend	−0.0167 ***	($-5.78\times 10^{14}$)
SL × Time trend	−0.0105 ***	($-3.57\times 10^{14}$)
SN × Time trend	−0.00858 ***	($-2.98\times 10^{14}$)
ST × Time trend	−0.00943 ***	($-3.19\times 10^{14}$)
TH × Time trend	−0.00311 ***	($-1.08\times 10^{14}$)
Constant	0.333 ***	(26.80)
Observations	320	
$R^{2}$	0.424	
$R^{2}$ adjusted	0.360	

*t* statistics in parentheses; two and three asterisks correspond to p<0.01 and p<0.01, respectively.

To completely interpret the results, consider the federal state Bremen. The constant 0.333 can be interpreted as the starting value of the share $s_{\mathrm{HB},t}$ for the federal state. Week t=1 in our sample is the last week of December 2020. Subsequently, i.e., in calender weeks t∈[1,2,…,19] of 2021, that share increases by 0.0108 per week. For weeks 14–19, i.e., the calender weeks starting on 5 April 2021, the level of $s_{\mathrm{HB},t}$ increases further by 0.116. Thus, in the last week, the average effect assigned to Bremen would be 0.6542.

Whereas we observe a clear time trend in the data for Bremen and, for instance, for Thuringia (TH) (see Figure 3 in the main text), other states do not exhibit such an obvious trend. In Equation (A1), we, therefore, still allow for a general time trend but do not impose such a trend for each federal state. In contrast, as a next step we approach the problem differently by allowing at the same time the analysis of whether the potential effect of the integration of doctor’s offices into Germany’s vaccination campaign differs between federal states.

(A2) $$s_{i,t}=\alpha +\beta t+\rho D_{\mathrm{April}\;5}+\eta_{i}D_{i}\times D_{\mathrm{April}\;5}+D_{i}+\epsilon_{i,t}.$$

In Equation (A2), $s_{i,t}$ is explained by a time trend *t*, the variable $D_{\mathrm{April}\;5}$ that takes the value 1 for the period after 5 April 2021, and 0 otherwise as well as a federal state specific fixed effect $D_{i}$. The federal state dummy $D_{i}$ is also interacted with $D_{\mathrm{April}\;5}$ to investigate whether a change in $s_{i,t}$ potentially triggered by the integration of doctor’s offices into the vaccination campaign differs between federal states. This approach enables an investigation of whether the integration of doctor’s offices into the vaccination campaign potentially improved efficiency of the vaccination campaign on average and whether this effects differs between federal states at the same time. The interaction term $D_{i}\times D_{\mathrm{April}\;5}$ is defined such that the results of each federal state have to be interpreted relative to Bremen. The results of the regression are presented in Table A4.

**Table A4:** Output table for Equation (A2).

	Dep. Var. $s_{i,t}$	
$D_{\mathrm{April}\;5}$	0.247 ***	(8.87)
$D_{\mathrm{April}\;5}$ × BB	−0.206 ***	($-8.18\times 10^{13}$)
$D_{\mathrm{April}\;5}$ × BE	−0.185 ***	($-7.26\times 10^{13}$)
$D_{\mathrm{April}\;5}$ × BW	−0.0927 ***	($-3.74\times 10^{13}$)
$D_{\mathrm{April}\;5}$ × BY	−0.181 ***	($-4.04\times 10^{13}$)
$D_{\mathrm{April}\;5}$ × HE	−0.124 ***	($-4.77\times 10^{13}$)
$D_{\mathrm{April}\;5}$ × HH	−0.158 ***	($-5.35\times 10^{13}$)
$D_{\mathrm{April}\;5}$ × MV	−0.142 ***	($-5.20\times 10^{13}$)
$D_{\mathrm{April}\;5}$ × NI	−0.0713 ***	($-2.87\times 10^{13}$)
$D_{\mathrm{April}\;5}$ × NW	0.0120 ***	($4.82\times 10^{12}$)
$D_{\mathrm{April}\;5}$ × RP	−0.186 ***	($-7.37\times 10^{13}$)
$D_{\mathrm{April}\;5}$ × SH	−0.183 ***	($-6.61\times 10^{13}$)
$D_{\mathrm{April}\;5}$ × SL	−0.198 ***	($-7.99\times 10^{13}$)
$D_{\mathrm{April}\;5}$ × SN	−0.136 ***	($-5.44\times 10^{13}$)
$D_{\mathrm{April}\;5}$ × ST	−0.108 ***	($-4.35\times 10^{13}$)
$D_{\mathrm{April}\;5}$ × TH	−0.147 ***	($-5.93\times 10^{13}$)
Time trend	0.000941	(0.34)
Constant	0.333 ***	(14.03)
Observations	320	
$R^{2}$	0.40	
$R^{2}$ adjusted	0.332	

*t* statistics in parentheses; three asterisks correspond to p<0.01.

One can see that the coefficient of the dummy $D_{\mathrm{April}\;5}$ is significant at the 0.1%-level. The coefficient of 0.247 reported in Table A4 implies that, all else equal, in the period after 5 April, 24.7% moreout of 100 available doses in a given week were vaccinated on average, or, in other words, 24.7% less doses were held back as reserves in Bremen. In interpreting the results, the potential effect of the integration of doctor’s offices into the vaccination campaign is a jump in $s_{i,t}$ by 24.7% for Bremen. That jump is lower for all other federal states except for Northrhine-Westphalia, where the jump is 0.247+0.0120=0.259. The lowest increase occurs in Brandenburg (BB) where the apparent effect of the integration of doctor’s offices into the vaccination campaign is 0.247−0.206=0.041.

A more detailed discussion on the differences between Regressions (A1) and (A2) is appropriate. The main difference is that (A1) allows the time trend to vary between federal states whereas (A2) allows the effect of the dummy $D_{\mathrm{April}\;5}$ to be federal state-specific. In contrast to (A1), in (A2) all differences in the time dimension between federal states is absorbed by the interaction term $D_{i}\times D_{\mathrm{April}\;5}$ so that the time trend eventually becomes insignificant. Based on a graphical analysis of Figure 3 in the main text one cannot clearly state which approach is more appropriate. For instance, Lower-Saxony (NI) and Hesse (HE) show similar patterns after 5 April, however, Lower-Saxony shows a clearer time trend. This indicates that Equation (A1) appears to be a more suitable approach to identify differences in the time dimension between federal states. On the other hand, comparing Bremen and Lower-Saxony, one can see that Bremen has a more pronounced effect of $D_{\mathrm{April}\;5}$. One could argue that there is a more visible structural break in Bremen than in Lower-Saxony. In that case, Equation (A2) seems more appropriate to identify differences in the time dimension. Based on these observations, it seems appropriate to discuss both variations. In any case, both estimations indicate structural breaks after 5 April, so that the results presented here confirm the findings outlined in the main text.

Finally, it can be analyzed which federal states show a time trend and for whom a structural break after 5 April can be diagnosed. In doing so, we estimate the following regression for each federal state:

(A3) $$s_{i,t}=\alpha_{i}+\beta_{i}t+\rho_{i}D_{\mathrm{April}\;5}+\gamma_{i}D_{\mathrm{April}\;5}\times t.$$

In Equation (A3), we test whether each federal state has a time trend, a structural break after April 5 and whether the time trend changes after April 5. The results are presented in Table A5.

**Table A5:** Time trend and structural break after 5 April 2021, and the interaction between the time trend and the structural break in $s_{i,t}$ for each federal state. The number of stars indicates the *p*-values of the respective t-statistics. In contrast to the previous analyses, *** indicates the p≤0.01, ** p≤0.05 and * p≤0.1.

Federal State	Time Trend (*t*)	Structural Break ($D_{\mathrm{April}\;5}$)	Interaction ($D_{\mathrm{April}\;5}\times t$)
BB	none	positive **	none
BE	none	positive ***	negative ***
BW	none	positive **	none
BY	none	positive *	none
HB	none	positive ***	negative **
HE	none	none	none
HH	none	positive *	none
MV	negative ***	none	positive **
NI	none	none	none
NW	none	positive ***	none
RP	none	none	none
SH	none	none	none
SL	none	none	none
SN	none	positive ***	negative ***
ST	none	positive ***	negative **
TH	positive **	positive ***	negative ***

Table A5 shows that 10 out of 16 federal states exhibit a structural break after 5 April 2021. That structural break is positive, i.e., an upward jump in $s_{i,t}$ is observed. However, starting from that higher level, one can see that in 5 of these federal states (HB, BE, SN, ST, TH) the shares $s_{i,t}$ start to decline again, which follows from the negative sign of the coefficient $\gamma_{i}$ of the interaction term $D_{\mathrm{April}\;5}\times t$. This indicates that in these federal states there was a strong initial impetus on $s_{i,t}$ at the beginning of 5 April, which began to level out afterwards. In the remaining 5 federal states (BB, BW, BY, HH, NW) the shares remain at a higher level. For Mecklenburg Western Pomerania (MV), we do not find a structural break, however, a negative time trend is found with an opposing positive trend after 5 April (positive coefficient of the interaction term). No effect of the integration of doctor’s offices into the vaccination campaign is found for 5 federal states (HE, NI, RP, SH, SL).

As a further robustness check, a Zivot-Andrews structural break test [38] was performed. This can be seen as a conservative approach because the test identifies endogenously at most one structural break. Based on the data, a structural break was identified in calender weeks 13 and 14 (i.e., the weeks around 5 April) in 6 federal states (including, in particular, Bremen and Northrhine-Westphalia) despite the scarcity of observations. These findings are consistent with the interpretation outlined in the main text that the identified effect is driven by those federal states where the integration of general practitioners into the vaccination campaign had a particularly strong effect.
